# Iron-Amended *Pleurotus citrinopileatus* Residue Composting: Lignocellulose Degradation and Microbial Functional Potential

**DOI:** 10.3390/foods15173158

**Published:** 2026-09-06

**Authors:** Wang Li, Ruiting Guo, Zhichao Zu, Shuai Xu, Guangjie Zhang, Yueting Dai, Yu Li

**Affiliations:** Engineering Research Center of Edible and Medicinal Fungi, Ministry of Education, Jilin Agricultural University, Changchun 130118, China; liwang@mails.jlau.edu.cn (W.L.); 18946755462@163.com (R.G.); zelzuzhichao@163.com (Z.Z.); xushuai@jlau.edu.cn (S.X.); 18763828272@163.com (G.Z.); yuli966@126.com (Y.L.)

**Keywords:** waste valorization, magnetite, humification, metagenomics, carbohydrate-active enzymes, carbon metabolism, compost maturity

## Abstract

Spent mushroom substrate (SMS) from *Pleurotus citrinopileatus* cultivation is a lignocellulose-rich by-product whose efficient composting is hindered by lignocellulose recalcitrance and slow humification. This study compared the effects of Fe_3_O_4_-based magnetic powder (MP), Fe^0^ beads (IB), and Fe_2_O_3_-rich crude iron ore (RI) with those of an unamended control during 30-day aerobic co-composting with chicken manure. Physicochemical characterization and metagenomic sequencing were combined to assess compost maturity, lignocellulose degradation, humic substance transformation, microbial succession, and functional potential. MP achieved the most balanced overall performance, exhibiting the highest total lignocellulose degradation (59.48%), the lowest final organic matter content (357.70 g/kg), a final C/N ratio of 13.04, and the highest germination index (151.50%). RI achieved the highest hemicellulose degradation and final contents of humic acid and total humic substances, whereas IB promoted rapid initial heating and relatively high cellulose degradation but caused greater physicochemical fluctuations. Metagenomic analyses revealed amendment-specific microbial succession and distinct CAZy-annotated gene and MetaCyc pathway profiles associated with lignocellulose transformation and carbon metabolism. Overall, Fe_3_O_4_-based magnetic powder showed the greatest potential for simultaneously promoting lignocellulose degradation, organic matter transformation, and compost maturity, providing a practical basis for the sustainable valorization of edible mushroom industry by-products.

## 1. Introduction

The global edible mushroom industry has expanded rapidly in recent years, generating large amounts of spent mushroom substrate (SMS) as a major organic by-product. Global edible mushroom production has been projected to reach 20.84 million tons in 2026, while annual SMS production exceeds 60 million tons and has been estimated to reach approximately 104 million tons in the same year [1]. SMS consists mainly of fungal residues and lignocellulose-rich agricultural materials, such as straw, sawdust, and wheat bran, and contains abundant organic carbon, nutrients, and trace minerals [2,3]. Therefore, efficient SMS utilization is important for biomass recycling and the sustainable development of the edible mushroom industry [4].

*Pleurotus citrinopileatus* is an edible and medicinal mushroom widely cultivated in China. Its short cultivation cycle, rapid growth, and strong environmental adaptability have led to increased production and a corresponding rise in *P. citrinopileatus* SMS [5]. Improper disposal of *P. citrinopileatus* SMS not only wastes biomass resources but may also cause soil and water pollution. Composting is an effective approach for the resource recovery and environmentally sound utilization of SMS, converting it into a mature organic amendment through microbially mediated organic matter transformation [6,7]. Co-composting with chicken manure provides a protein-rich nitrogen source that helps adjust the initial C/N ratio and supports microbial succession and humification during SMS composting [8]. However, the compact and recalcitrant lignocellulosic matrix of *P. citrinopileatus* SMS often results in prolonged composting periods, slow organic matter transformation, and insufficient humification. Compost maturity depends on coordinated lignocellulose degradation and the formation of humic substances. Humic substances (HS), including humic acid (HA), fulvic acid (FA), and humin (HM), are important indicators of humification and compost maturity. Thus, improving the composting microenvironment and supporting microbial communities with lignocellulose-degrading potential may promote lignocellulose conversion, humification, and SMS resource recovery [9,10,11].

Iron-based additives have been widely used to improve composting efficiency and influence microbially mediated organic matter transformation [12]. Under suitable conditions, some iron-containing materials may also participate in redox reactions, including Fenton-like processes; however, the occurrence and intensity of these reactions depend strongly on iron speciation and environmental conditions [13]. Fe^0^-, Fe_3_O_4_-, and Fe_2_O_3_-based materials differ not only in their nominal iron oxidation states but also in mineral composition, particle properties, solubility, and surface reactivity, and may therefore exert amendment-specific effects on composting performance, organic matter transformation, and humification [14]. Although Fe^0^-, Fe_3_O_4_-, and Fe_2_O_3_-based amendments have been investigated in other composting systems, their comparative effects on lignocellulose fraction degradation, humic substance transformation, microbial succession, and microbial functional potential during *P. citrinopileatus* SMS composting remain poorly understood.

Carbohydrate-active enzymes (CAZymes) provide an important molecular basis for microbial lignocellulose degradation, and the abundance of CAZyme-encoding genes reflects the functional potential for carbohydrate transformation during composting. Combining metagenomic sequencing with CAZy and MetaCyc annotations enables a gene-level evaluation of microbial functional potential and carbon metabolism-related profiles during composting [15].

In this study, *P. citrinopileatus* SMS and chicken manure were aerobically co-composted for 30 days with Fe_2_O_3_-rich crude iron ore, Fe_3_O_4_-based magnetic powder, or Fe^0^ beads as amendments. The novelty of this study lies in the controlled, side-by-side comparison of these three compositionally distinct iron-based amendments within the same SMS composting system, integrating composting performance, lignocellulose degradation, humic substance transformation, microbial succession, and CAZy- and MetaCyc-annotated functional profiles. The findings provide a comparative basis for selecting suitable iron-based amendments for the sustainable composting and valorization of edible mushroom industry by-products.

## 2. Materials and Methods

### 2.1. Composting Materials

*P. citrinopileatus* SMS and chicken manure were used as the principal organic feedstocks [8,9]. The SMS was obtained after laboratory cultivation of *P. citrinopileatus* and had a moisture content of approximately 60%. On a dry-weight basis, the original cultivation substrate consisted of 60% sawdust, 30% corn cob, 8% wheat bran, and 2% lime. Chicken manure was obtained from Jilin Agricultural University. Three commercially available iron-based materials were used: Fe_2_O_3_-rich crude iron ore, Fe_3_O_4_-based magnetic powder, and Fe^0^ beads [12,14]. All three materials were purchased from a local hardware supplier in Changchun, Jilin Province, China.

### 2.2. Experimental Design

Four treatments were established: an unamended control (CK), an Fe_2_O_3_-rich crude iron ore treatment (RI), an Fe_3_O_4_-based magnetic powder treatment (MP), and an Fe^0^ bead treatment (IB). The treatment compositions and basic characteristics of the iron-based amendments are summarized in Table 1.

The composting experiment was conducted at the Mushroom–Vegetable Base of Jilin Agricultural University, Changchun, China, using 100 L composting reactors. Each treatment comprised three independent reactors. *P. citrinopileatus* SMS and chicken manure were mixed at a dry-weight ratio of 3:1. In the RI, MP, and IB treatments, the corresponding iron-based amendment was added at 3% of the total dry matter, whereas CK received no iron-based amendment. Each reactor contained 50 kg of wet material, and the initial moisture content was adjusted to 60%. Composting was conducted for 30 days, and the materials were turned every 5 days to improve aeration and homogenization.

### 2.3. Compost Sample Collection and Pretreatment

Samples were collected at 5-day intervals on days 0, 5, 10, 15, 20, 25, and 30. For each reactor, approximately 100 g of material was collected from each of the upper, middle, and lower layers and thoroughly combined to form one composite sample.

Each composite sample was divided into three portions. The first portion was air-dried, ground, and passed through a 0.25 mm sieve for the determination of organic matter (OM), total nitrogen (TN), total carbon (TC), available potassium (AK), available phosphorus (AP), and lignocellulose components. The second portion was stored at 4 °C for the determination of pH, electrical conductivity (EC), NH_4_^+^-N, NO_3_^−^-N, and humic substances (HS), including humic acid (HA), fulvic acid (FA), and humin (HM). The third portion was immediately frozen at −80 °C for subsequent metagenomic DNA extraction. Samples collected on days 0, 15, and 30 were selected for metagenomic sequencing and subsequent analyses of microbial community composition and functional gene profiles.

### 2.4. Analytical Methods

#### 2.4.1. Physicochemical Measurements and Germination Index

During the 30-day aerobic composting process, temperature was monitored daily at 14:00 in the upper, middle, and lower layers of each reactor to account for spatial heterogeneity. Temperature, pH, and EC were determined in triplicate, and the results are presented as mean ± SE. Temperature was measured using a JRYS digital thermometer (Kedibo, Hengshui, China). The pH and EC of the compost extracts were determined using a PH200 pH meter (Sanliang, Dongguan, China) and a CT-2 conductivity meter (LICHEN, Shanghai, China), respectively.

The germination index (GI) of cucumber seeds was used to evaluate compost phytotoxicity. Compost samples were mixed with deionized water at a ratio of 1:10 (*w*/*v*), shaken at 25 °C and 180 r/min for 1 h, and centrifuged at 4500 r/min for 10 min to obtain extracts. Ten cucumber seeds were placed in 9 cm Petri dishes containing two filter papers and 10 mL of extract, with deionized water used as the control. All dishes were incubated at 25 °C in the dark for 72 h. GI was calculated as follows:

$$\mathrm{GI}\left(\%\right)=\frac{R\;\times \;L}{R_{0}\;\times \;L_{0}}\times 100\%$$

Note: R, germination rate of treatment; L, mean root length of treatment; R_0_, germination rate of control; L_0_, mean root length of control.

#### 2.4.2. Determination of Physicochemical Properties

NO_3_^−^–N and NH_4_^+^–N were determined using commercial assay kits (Suzhou Michy Biomedical Technology Co., Ltd., Suzhou, China) according to the manufacturer’s instructions [16], based on salicylic acid colorimetry at 410 nm and indophenol blue colorimetry at 625 nm, respectively. TN was determined by the modified Kjeldahl method with reference to HJ 717-2014 [17]. OC was measured using a Tyurin-type K_2_Cr_2_O_7_–H_2_SO_4_ external-heating oxidation method (170–180 °C, 5 min), followed by FeSO_4_ titration, as described in NY/T 1121.6-2006 [18]. OM was calculated as OC × 1.724, assuming that OM contains approximately 58% carbon [19]. TC was determined by high-temperature dry combustion and non-dispersive infrared detection using a multi N/C 2100S analyzer equipped with a solid combustion module (Analytik Jena GmbH, Jena, Germany), with reference to ISO 10694:1995 [20].

Available phosphorus (AP) was determined using an Olsen-type extraction procedure [21]. All compost samples were extracted with 0.5 mol L^−1^ NaHCO_3_ at a sample-to-extractant ratio of 1:20 (*w*/*v*) for 30 min at 25 ± 1 °C, and AP was subsequently quantified by molybdenum–antimony colorimetry at 880 nm [21]. AK was extracted with 1 mol L^−1^ NH_4_OAc [21] and measured by ICP-OES with reference to ISO 22036:2024 [22].

Humic fractions were determined using a modified Na_4_P_2_O_7_–NaOH extraction and dichromate oxidation method, with reference to LY/T 1238-1999 [23]. Briefly, 0.1 g of air-dried sample was extracted with 25 mL of 0.1 mol L^−1^ Na_4_P_2_O_7_–0.1 mol L^−1^ NaOH, shaken for 5 min, heated in a boiling-water bath for 1 h, and clarified by filtration [24]. Total extractable humic carbon (HA-C + FA-C) was measured in a 5 mL aliquot by K_2_Cr_2_O_7_ external-heating oxidation after neutralization to pH 7. For HA separation, another 5 mL aliquot was acidified to pH 2–3 with 0.5 mol L^−1^ H_2_SO_4_, maintained at 80 °C for 30 min, and left overnight. The HA precipitate was washed with 0.05 mol L^−1^ H_2_SO_4_, redissolved in hot 0.05 mol L^−1^ NaOH, and quantified by the same oxidation method. FA-C and humin-C were calculated by difference according to LY/T 1238-1999 [23].

Lignin, cellulose, and hemicellulose were determined using Solarbio assay kits BC4205, BC4285, and BC4445, respectively (Beijing Solarbio Science & Technology Co., Ltd., Beijing, China), according to the manufacturer’s instructions [25,26]. Lignin was measured by the acetyl bromide method at 280 nm, cellulose by anthrone colorimetry at 620 nm, and hemicellulose by the 3,5-dinitrosalicylic acid (DNS) method at 540 nm.

#### 2.4.3. DNA Extraction and Metagenomic Sequencing

Metagenomic DNA was extracted from 0.5 g of each compost sample using a FastPure Soil DNA Isolation Kit (MJYH, Shanghai, China) according to the manufacturer’s instructions [27]. DNA integrity was assessed by 1% agarose gel electrophoresis, and DNA concentration and purity were evaluated using a NanoDrop 2000 spectrophotometer (Thermo Fisher Scientific, Wilmington, DE, USA). The qualified DNA was fragmented to approximately 350 bp, and paired-end libraries were constructed using the NEXTFLEX^®^ Rapid DNA-Seq Kit (Bioo Scientific, Austin, TX, USA). Metagenomic sequencing was performed on an Illumina NovaSeq X Plus platform (Illumina Inc., San Diego, CA, USA) to generate PE150 reads (2 × 150 bp).

#### 2.4.4. Sequence Processing and Gene Abundance Calculation

The four treatments (CK, RI, MP, and IB) were sampled on days 0, 15, and 30, with three biological replicates per treatment and sampling time, yielding 36 metagenomic samples. Raw paired-end reads were quality-filtered using fastp v0.23.0. The high-quality reads were assembled using MEGAHIT v1.1.2, and open reading frames were predicted from the assembled contigs. The predicted genes were clustered using CD-HIT v4.6.1 to construct a nonredundant gene catalog. High-quality reads from each sample were mapped to the nonredundant gene catalog using SOAPaligner v2.21 at 95% sequence identity. The relative abundance of each gene was normalized as reads per kilobase per million mapped reads (RPKM), thereby accounting for differences in gene length and sequencing depth [28,29]. Functional-category abundance was calculated by summing the RPKM values of genes assigned to the corresponding category. RPKM values were interpreted as normalized relative functional-gene abundances within the present dataset rather than as measures of RNA expression or absolute gene copy number. Using the Majorbio Metagenomic Cloud Analysis Pipeline (https://www.majorbio.com/), bioinformatics analyses (quality control, assembly, gene prediction, annotation, diversity, and interaction analysis) were conducted to reveal microbial community structure and functional metabolic profiles across treatments and composting stages [30].

#### 2.4.5. Functional Annotation of Carbohydrate-Active Enzymes and Metabolic Pathways

To characterize the gene-level potential for carbohydrate degradation and carbon metabolism during aerobic composting, high-quality metagenomic data were functionally annotated against the CAZy database [31] and the MetaCyc database. For carbohydrate-active enzyme annotation, the predicted protein sequences in the nonredundant gene catalog were searched against the CAZy v12 database using hmmscan implemented in HMMER v3.1b2, using an E-value cutoff of 1 × 10^−5^. The abundances of individual CAZy families and functional classes were obtained by summing the RPKM values of their annotated genes. Metabolic pathways were annotated against the MetaCyc database using HUMAnN v3.0.1, using an E-value cutoff of 1 × 10^−5^. The resulting CAZy and MetaCyc profiles were used to compare the relative functional potential of the microbial communities among treatments and composting stages.

## 3. Results

### 3.1. Changes in Physicochemical Properties and Compost Maturity During Composting

Temperature, pH, EC, GI, available nutrients, nitrogen forms, TN, TC, and the C/N ratio were monitored during the 30-day composting period to compare the effects of the three iron-based amendments on the composting environment and maturity. Based on temperature dynamics, the composting process was divided into heating, thermophilic, cooling, and maturation phases [32,33].

#### 3.1.1. Visual Changes in Compost Appearance

**Figure 1:**
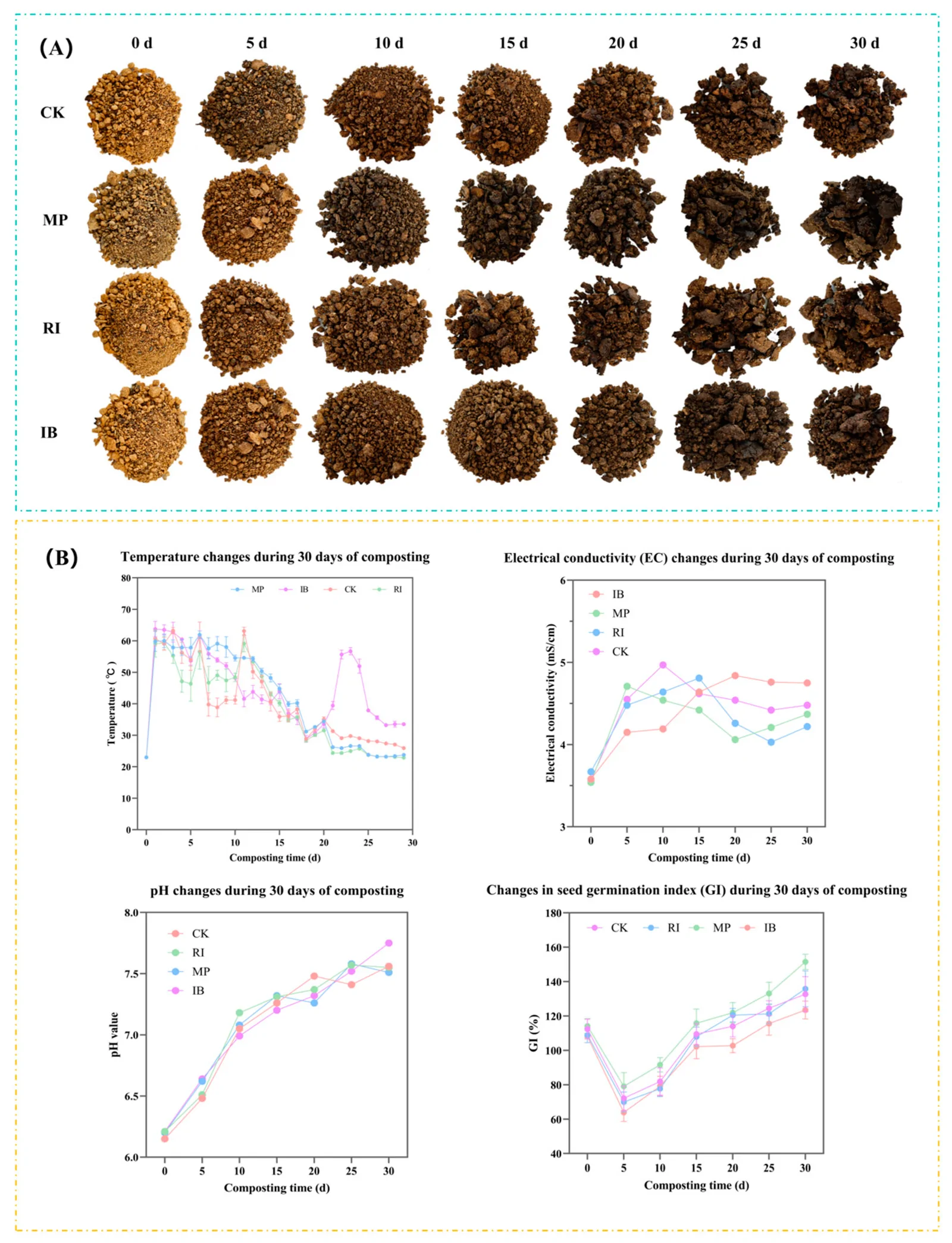
Changes in compost appearance, physicochemical properties, and maturity indicators during 30-day composting under different treatments. (**A**) Visual appearance of compost samples collected on days 0, 5, 10, 15, 20, 25, and 30. (**B**) Temporal changes in temperature, EC, pH, and GI. Quantitative data are presented as the mean ± SE of three independent replicates (*n* = 3).

During the 30-day composting period, all treatments showed progressive darkening and a gradual loss of recognizable structures of the original SMS particles. Fine particles generated through substrate fragmentation and degradation subsequently coalesced into aggregates. Compared to CK, the iron-amended treatments exhibited more pronounced changes in color and aggregate morphology. MP formed the darkest and most compact aggregates at the final stage, whereas RI and IB showed varying degrees of darkening, substrate fragmentation, and secondary aggregation. These visual changes were broadly consistent with the subsequent results for lignocellulose degradation and humification (Figure 1A).

#### 3.1.2. Changes in Temperature, pH, EC, and GI

Temperature, pH, EC, and GI showed treatment-dependent temporal patterns (Figure 1B). During the heating and thermophilic stages (0–15 d), temperature rose from approximately 23 °C to nearly 60 °C. IB showed the fastest initial heating, reaching 63.73 °C on day 1; RI showed delayed heating, whereas MP maintained temperatures above 55 °C for more than 3 days. EC increased from 3.52–3.66 to 4.19–4.97 mS/cm, and pH from 6.09–6.31 to 6.99–7.15. GI decreased in all treatments on day 5, with the lowest value in IB (63.92%) and a higher value in MP (79.11%), indicating treatment-dependent differences in early phytotoxicity. During the cooling and maturation stages (15–30 d), temperature declined to 23–30 °C. In MP, EC decreased to 4.21 mS/cm, pH reached 7.54, and GI increased from 115.90% on day 15 to 151.50% on day 30, indicating reduced phytotoxicity and increased compost maturity. IB showed a secondary temperature peak of 39.47 °C on day 21, while its GI increased from 102.80% on day 20 to 123.50% on day 30. Final GI followed MP (151.50%) > RI (135.80%) > CK (132.70%) > IB (123.50%). Overall, MP combined sustained thermophilic conditions with the highest final GI, whereas IB showed the fastest heating but the lowest final GI.

#### 3.1.3. Changes in AP, AK, NH_4_^+^-N, and NO_3_^−^-N Contents

**Figure 2:**
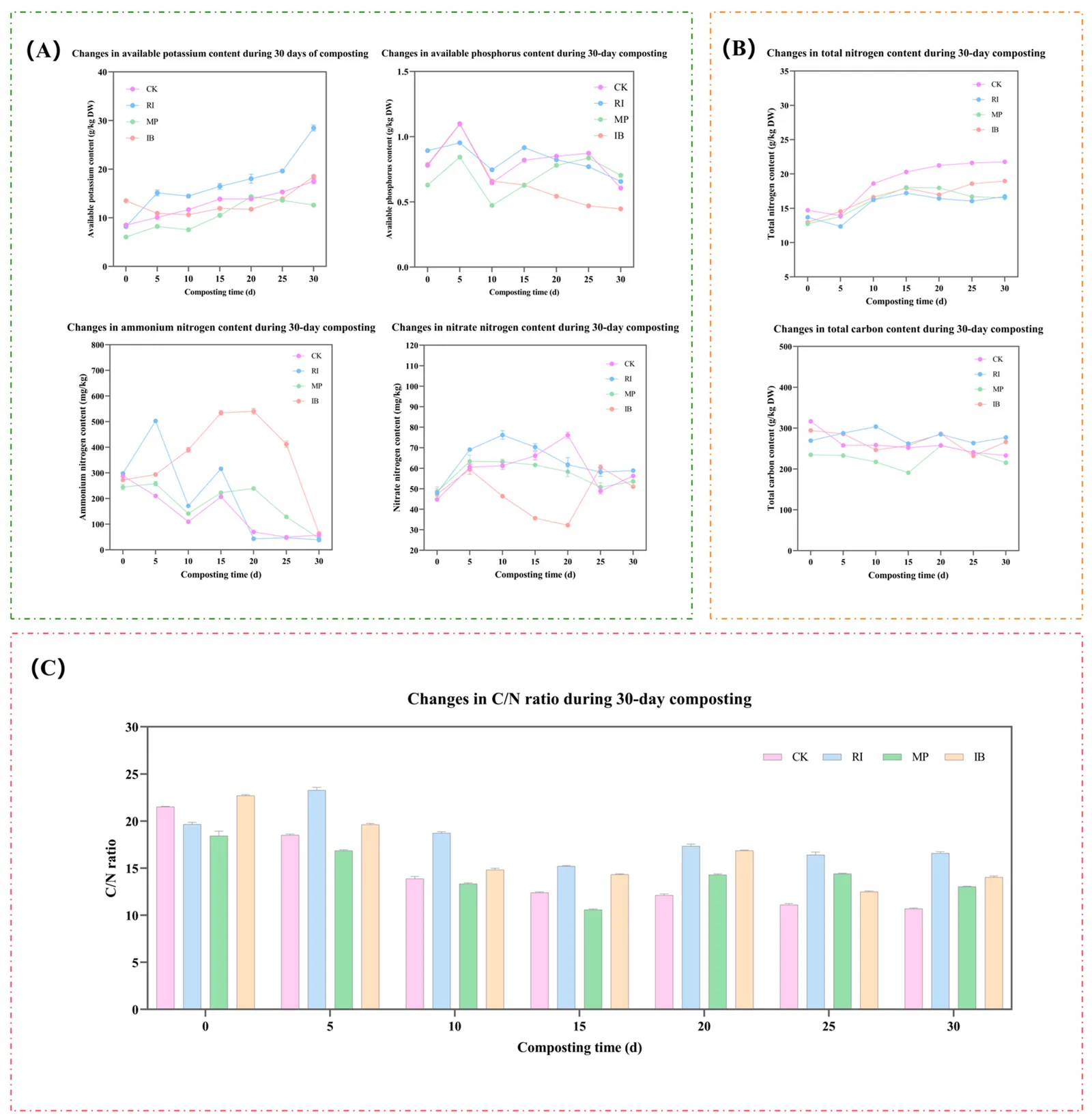
Changes in available nutrients, nitrogen forms, total carbon, and the C/N ratio during 30-day composting under different treatments. (**A**) Temporal changes in AK, AP, NH_4_^+^–N, and NO_3_^−^–N. (**B**) Temporal changes in TN and TC. (**C**) Temporal changes in the C/N ratio. Data are presented as the mean ± SE of three independent replicates (*n* = 3).

AP, AK, NH_4_^+^-N, and NO_3_^−^-N varied among treatments during composting (Figure 2A). During days 0–5, NH_4_^+^-N increased to 502.80 mg/kg in RI and 294.00 mg/kg in IB, exceeding CK (210.30 mg/kg). AP reached 1.10 g/kg in CK and IB but was lower in MP (0.84 g/kg), while AK reached 15.12 g/kg in RI and 8.22 g/kg in MP. During days 5–15, NH_4_^+^-N decreased in most treatments, reaching 141.30 mg/kg in MP, but remained high in IB (534.40 mg/kg). Meanwhile, NO_3_^−^-N in IB and AP in MP declined to 35.65 mg/kg and 0.47 g/kg, respectively, while AK in RI increased to 16.48 g/kg. During days 15–30, final NH_4_^+^-N contents in MP and RI were lower than in CK, whereas IB maintained relatively high levels through day 25. On day 30, AK reached 28.47 g/kg in RI but decreased to 12.62 g/kg in MP. Overall, the amendments differentially affected nitrogen forms and available nutrient dynamics.

#### 3.1.4. Changes in TN, TC, and C/N Ratio

TN, TC, and the C/N ratio reflected nitrogen retention, carbon mineralization, and compost maturity (Figure 2B,C). During days 0–5, TN decreased in CK (14.71 → 13.93 g/kg) and RI (13.70 → 12.35 g/kg) but increased in MP (12.74 → 13.81 g/kg) and IB (12.96 → 14.56 g/kg), showing treatment-dependent nitrogen transformation. TC declined in all treatments except RI, with the largest decrease in CK (316.80 → 258.00 g/kg). The C/N ratio decreased in MP (18.45 → 16.87) and CK (21.54 → 18.52) but increased to 23.30 in RI. During days 5–15, organic carbon transformation intensified as TN increased in all treatments and TC continued to decline, reaching the lowest level in MP. The C/N ratio in MP consequently declined to 10.59, indicating accelerated carbon–nitrogen conversion under Fe_3_O_4_. Changes in all three indicators slowed during days 15–30. On day 30, MP reached a final TN of 16.52 g/kg and TC of 215.50 g/kg, with a C/N ratio of 13.04, meeting maturity standards. Overall, the sharp C/N decline in MP indicated faster carbon–nitrogen conversion and compost maturation.

### 3.2. Changes in Organic Matter Transformation and Lignocellulose Degradation During Composting

#### 3.2.1. Changes in OM and Humic Fraction Contents

**Figure 3:**
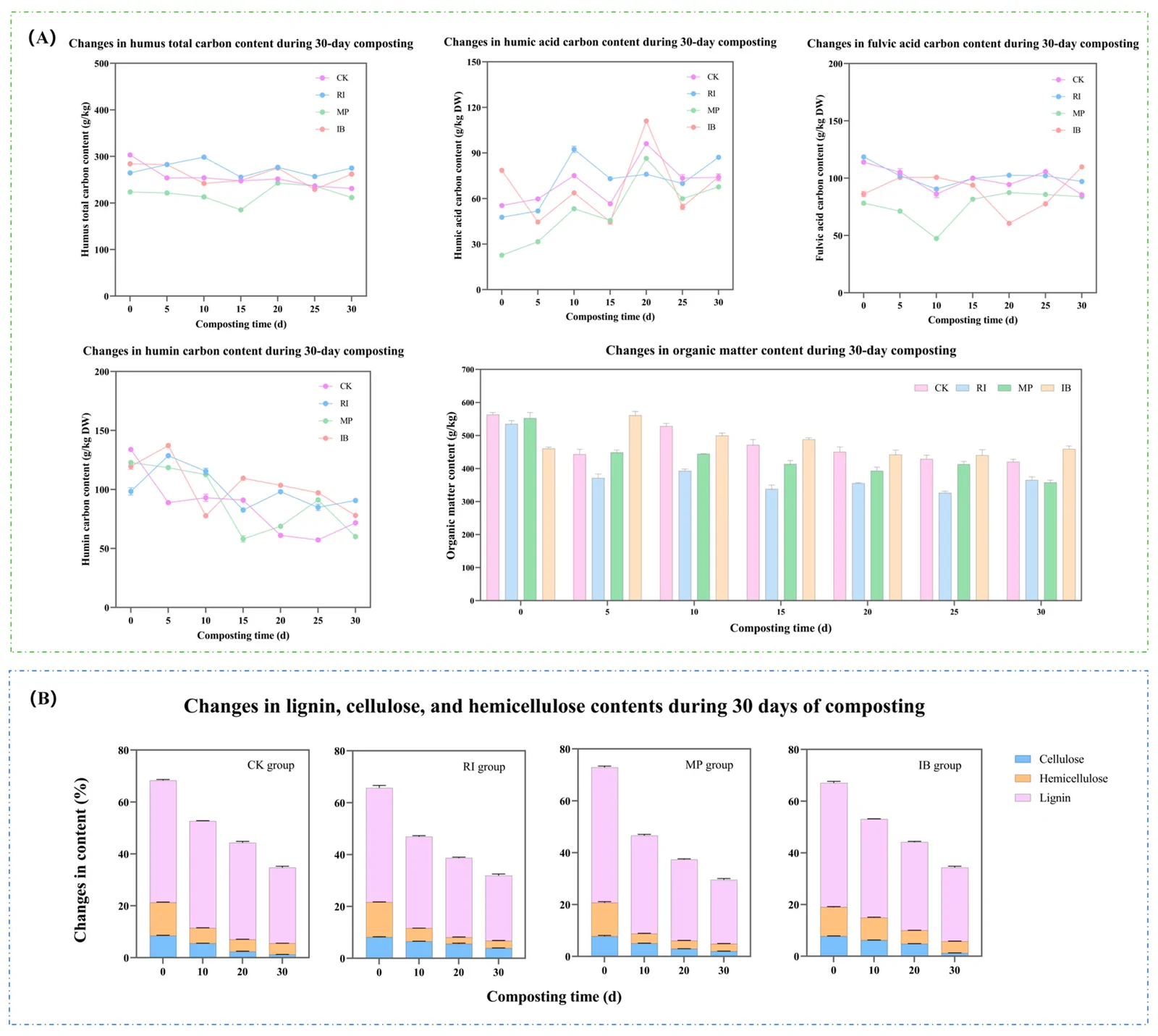
Changes in OM, humic-fraction carbon, and lignocellulosic component contents during 30 days of composting under different treatments. (**A**) OM and humic-fraction carbon contents. (**B**) Lignin, cellulose, and hemicellulose contents. Data in panels (**A**,**B**) are presented as the mean ± SE of three independent replicates (*n* = 3).

Changes in OM and humic fractions reflected the progression from organic matter mineralization to humus formation during composting (Figure 3A). During days 0–5, OM decreased to 443.10, 371.30, and 448.60 g/kg in CK, RI, and MP, respectively, but increased to 560.70 g/kg in IB. Meanwhile, HA increased in CK (55.40 → 59.78 g/kg) and MP (22.66 → 31.59 g/kg), marking the initiation of humification. During days 5–15, OM further decreased to 338.10 g/kg in RI and 413.40 g/kg in MP, while HA increased markedly in RI (51.94 → 73.07 g/kg) and MP (31.59 → 45.52 g/kg), accompanied by HM reduction. During days 15–25, OM degradation slowed, whereas humic fractions continued to transform, with HS in MP increasing from 185.40 to 236.90 g/kg. On day 30, final OM followed the order MP (357.70 g/kg) < RI (365.00 g/kg) < CK (420.10 g/kg) < IB (458.80 g/kg). RI showed the highest HS (274.90 g/kg) and HA (87.12 g/kg), whereas MP showed the greatest HM reduction (122.80 → 60.11 g/kg). Thus, RI favored HA accumulation, while MP enhanced OM depletion and HM transformation.

#### 3.2.2. Changes in Lignocellulose Degradation

Lignin, cellulose, and hemicellulose declined throughout composting, with treatment-specific patterns related to compost maturation and carbon transformation (Figure 3B and Table 2) [34]. MP showed the highest lignin degradation (52.69%) versus CK (38.01%), IB (40.52%), and RI (42.98%). Cellulose degradation was higher in CK (85.55%) and IB (84.11%) than in MP (74.42%), whereas hemicellulose degradation was highest in RI (78.96%), followed by MP (77.88%). Despite these differences, MP achieved the highest total lignocellulose degradation (59.48%), compared to CK (49.18%), IB (48.82%), and RI (51.45%).

Iron-based materials differentially affected lignocellulose fractions. Although Fe_3_O_4_ did not maximize the degradation of every component, it produced the most balanced overall conversion, potentially due to relatively stable redox reactivity and enrichment of lignocellulose-degrading microorganisms. Among the iron-amended treatments, Fe^0^ favored cellulose degradation but yielded less balanced total conversion, whereas Fe_2_O_3_ preferentially enhanced hemicellulose degradation. Thus, Fe_3_O_4_ most effectively coordinated lignocellulose conversion during SMS composting.

### 3.3. Metagenomic Analysis of Microbial Community Succession

**Figure 4:**
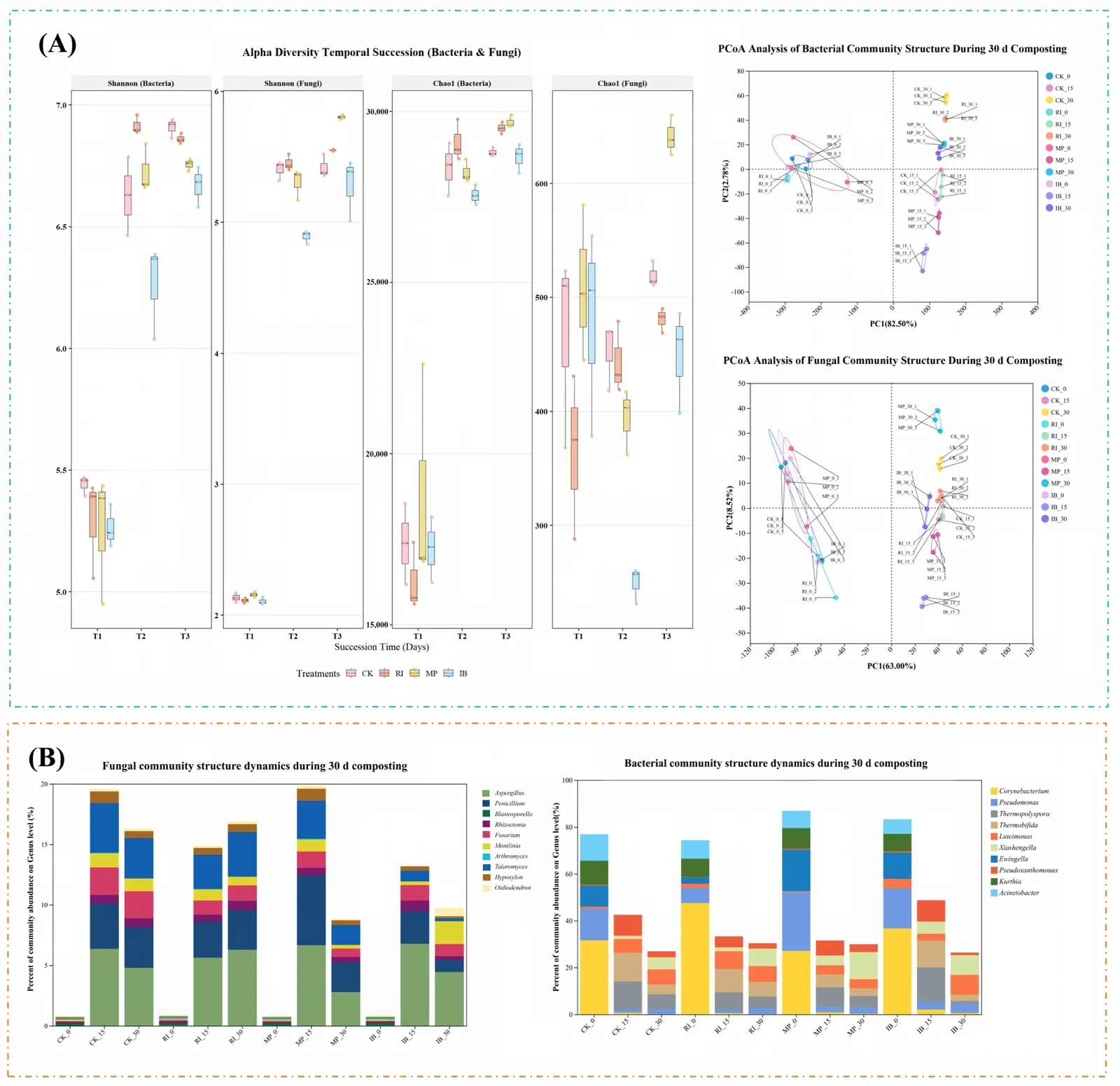
Diversity and community succession of bacterial and fungal communities during 30-day composting under different treatments. (**A**) Temporal changes in α-diversity indices calculated separately for bacterial and fungal communities, including the Shannon and Chao1 indices (**left**), and principal coordinate analysis (PCoA) of bacterial and fungal β-diversity based on genus-level community composition (**right**). T1, T2, and T3 represent samples collected on days 0, 15, and 30, respectively. (**B**) Stacked bar charts showing the temporal succession of dominant fungal genera (**left**) and bacterial genera (**right**).

Overall α-diversity, calculated from combined bacterial and fungal profiles, generally increased during composting. On day 30, Chao1 richness ranked MP > RI > CK > IB, whereas the Shannon index ranked CK > RI > MP > IB. In MP, the Shannon index increased markedly from day 0 to day 15 and remained nearly stable thereafter. Thus, MP supported the highest final microbial richness but lower community evenness than CK and RI (Table S1). To clarify kingdom-specific dynamics, bacterial and fungal α-diversity patterns are presented separately in Figure 4A.

PCoA clearly separated bacterial and fungal communities across composting stages and iron treatments (Figure 4A). PC1 explained 82.50% and 63.00% of bacterial and fungal variation, respectively, indicating that composting time and iron valence jointly shaped microbial succession. Treatment-specific enrichment of lignocellulose-degrading microorganisms was also observed.

At the phylum level, Pseudomonadota, Bacillota, Actinomycetota, and Bacteroidota dominated the bacterial communities and included major taxa associated with lignocellulose degradation. MP and IB showed stronger enrichment of Pseudomonadota and Bacillota than RI and CK [35] (Figure S1). At the genus level, the thermophilic polysaccharide-degrading genus *Thermopolyspora* peaked in IB_15 (0.0734), whereas MP maintained a relatively stable abundance during days 15–30 (0.0411–0.0257). *Thermobifida* peaked in CK_15 (0.0612) and remained relatively abundant in RI_30 (0.0315); *Luteimonas* and *Pseudoxanthomonas* were enriched in IB and MP, respectively [36,37]. Early-stage *Corynebacterium* and *Kurthia* declined rapidly, while MP maintained low *Pseudomonas* abundance at day 30, suggesting more stable succession potentially associated with Fe_3_O_4_ redox properties [38] (Figure 4B).

Ascomycota dominated the fungal communities (Figure S1). *Aspergillus* and *Penicillium* increased from initially low abundances (0.0002–0.0006) after the thermophilic stage, reaching 0.0666 and 0.0577, respectively, in MP at day 15. Conversely, *Blastosporella* declined from 0.0014 to 0.0016 and nearly disappeared after day 15, indicating poor thermophilic adaptation. At day 30, *Aspergillus* and *Penicillium* declined but remained detectable, and their transient enrichment in MP indicated enhanced fungal participation in lignocellulose transformation (Figure 4B).

Overall, MP maintained relatively stable bacterial and fungal degraders, including *Thermopolyspora*, *Luteimonas*, *Pseudoxanthomonas*, *Aspergillus*, and *Penicillium*, potentially associated with Fe_3_O_4_-related Fe^2+^/Fe^3+^ cycling. IB showed transient thermophilic enrichment but greater community fluctuation, whereas RI showed weaker sustained enrichment. These patterns were consistent with the higher overall lignocellulose conversion in MP.

### 3.4. Correlation Analysis Between Environmental Factors and Microbial Communities

**Figure 5:**
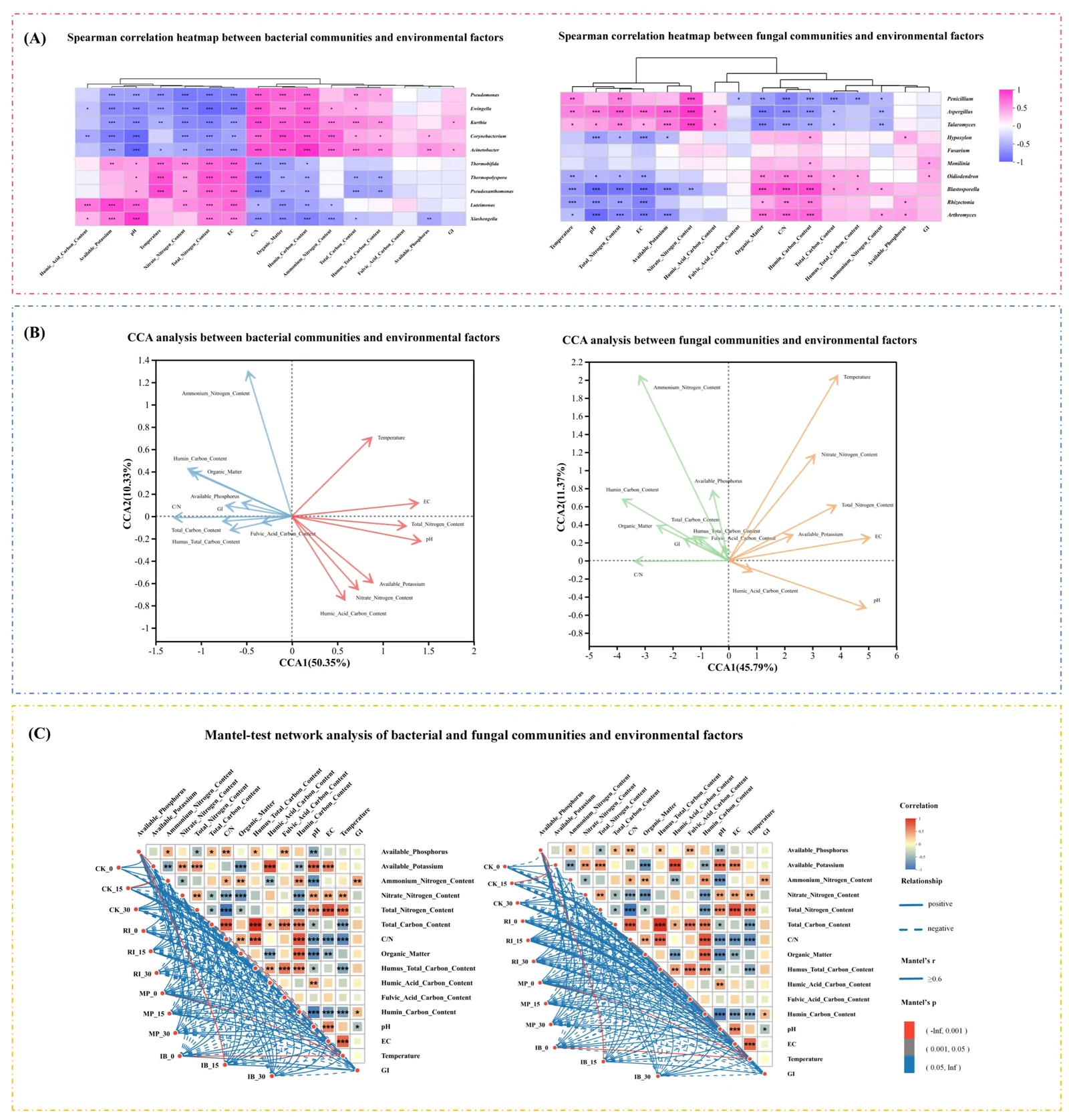
Multidimensional correlation analysis between microbial communities and environmental factors during 30-day composting. (**A**) Spearman correlation heatmaps of dominant bacterial and fungal genera with environmental factors. (**B**) CCA scatter plots of dominant bacterial and fungal genera associated with environmental factors. (**C**) Genus-level Mantel-test network analysis between bacterial (left)/fungal (right) communities and environmental factors. Lines represent correlations between microbial communities and environmental factors, and the internal heatmap shows pairwise correlations among environmental factors. Asterisks indicate significance levels: * 0.01 < *p* ≤ 0.05, ** 0.001 < *p* ≤ 0.01, *** *p* ≤ 0.001.

Spearman correlation analysis, canonical correspondence analysis (CCA), Mantel tests, and variation partitioning analysis (VPA) evaluated associations between microbial communities and environmental factors during composting. The Spearman heatmap (Figure 5A) identified TN, the C/N ratio, OM, NO_3_^−^-N, and NH_4_^+^-N as major factors associated with microbial succession. Thermophilic bacteria (*Thermobifida* and *Thermopolyspora*) and lignocellulose-degrading fungi (*Aspergillus* and *Penicillium*) were positively correlated with TN and NO_3_^−^-N but negatively correlated with OM and the C/N ratio, linking these taxa to nitrogen transformation and organic matter depletion. Conversely, NH_4_^+^-N was negatively associated with several functional degraders, suggesting that excessive ammonium may constrain microbial succession. CCA confirmed these patterns (Figure 5B). For bacteria, CCA1 and CCA2 explained 60.68% of the variation, with TN, NO_3_^−^-N, AK, and HA associated with succession and OM and the C/N ratio showing opposite effects. For fungi, the two axes explained 57.16% of the variation; *Aspergillus* and *Penicillium* were positively associated with thermophilic conditions but negatively associated with OM and the C/N ratio. Thus, bacterial and fungal decomposers showed similar environmental responses during lignocellulose degradation and nutrient transformation.

Mantel test network analysis (Figure 5C) showed few significant direct correlations between individual environmental factors and microbial communities but strong intercorrelations among environmental variables, indicating coupled effects during composting. The C/N ratio correlated negatively with TN (*r* = −0.845, *p* < 0.001) and positively with TC (*r* = 0.795, *p* < 0.001), defining a major carbon–nitrogen coupling axis. OM correlated negatively with AK, NO_3_^−^-N, and HA (*r* = −0.570, *p* < 0.001), whereas HA correlated positively with AK (*r* = 0.845, *p* < 0.001) and FA with HS (*r* = 0.675, *p* < 0.001), reflecting organic matter depletion and coordinated humic fraction transformation. Among physicochemical factors, pH correlated positively with TN (*r* = 0.600, *p* < 0.001), while temperature correlated negatively with the C/N ratio (*r* = −0.726, *p* < 0.001). VPA showed that environmental factors explained 79.96% of bacterial and 53.88% of fungal community variation (Figure S2), indicating stronger environmental filtering of bacteria. Thus, microbial succession reflected the combined effects of carbon–nitrogen balance, humic transformation, and physicochemical conditions.

### 3.5. Carbon Cycle Pathway Gene Enrichment and Regulation

**Figure 6:**
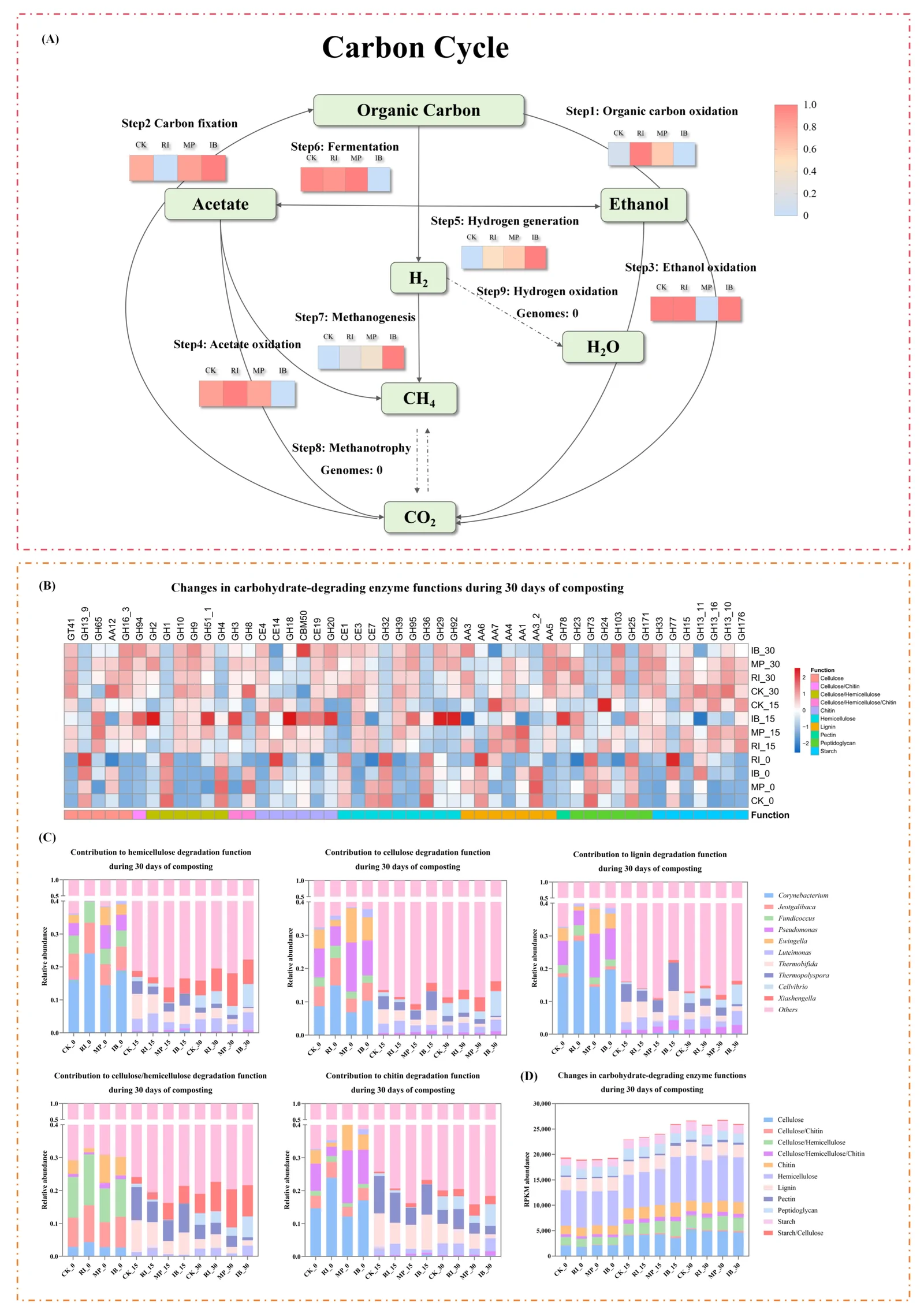
Heatmap of intergroup enrichment of carbon cycle pathway gene counts and comprehensive analysis of carbohydrate degradation-related functions and microbial contributions during 30 days of composting. (**A**) Mini heatmaps show gene counts across treatments with row-wise Min–Max normalization (0–1, red = high, blue = low). Detected pathways connected by solid lines, undetected by dashed lines; Steps 8 and 9 had 0 genes. (**B**) Heatmap of carbohydrate-degrading enzyme families: rows represent different families, columns represent compost samples, and color intensity indicates functional abundance. (**C**) Stacked bar chart of microbial genus contributions to degradation functions, with sample names on the x-axis and relative species abundance on the y-axis. (**D**) Stacked bar chart of major degradation functions: x-axis shows sample names, y-axis shows RPKM abundance, different colors denote distinct functions, and total bar heights reflect overall functional abundance.

MetaCyc annotation characterized the gene-level potential for microbial carbon metabolism during *P. citrinopileatus* SMS composting (Figure 6A) [39]. Seven pathway categories were detected: organic carbon oxidation, carbon fixation, ethanol oxidation, acetate oxidation, hydrogen production, fermentation, and methanogenesis. Genes related to methanotrophy and hydrogen oxidation were not detected; thus, methanogenesis was the only methane-related pathway identified under these composting conditions.

Normalized gene counts and the row-wise Min–Max heatmap showed that fermentation-related genes were more abundant than genes assigned to other carbon-transformation pathways, indicating fermentation as a major functional component. Enrichment differed among iron-valence treatments: MP (Fe_3_O_4_) showed the highest enrichment in fermentation; IB (Fe^0^) in hydrogen production, carbon fixation, and methanogenesis; and RI (Fe_2_O_3_) in organic carbon and acetate oxidation. CK showed weaker and less specific enrichment than the iron-amended treatments. Thus, iron amendments differentially shaped carbon-pathway gene abundance, providing pathway-level evidence for iron-mediated regulation of compost carbon transformation.

### 3.6. Carbon Metabolic Pathway Analysis Based on CAZy Families

Based on metagenomic sequencing and CAZy annotation, CAZyme-encoding gene abundance, estimated microbial contributions, and carbohydrate-degradation profiles were compared among CK and the Fe^0^-, Fe_3_O_4_-, and Fe_2_O_3_-based treatments during *P. citrinopileatus* SMS composting. The results revealed iron-valence-associated differences in microbial carbohydrate-degradation potential, linking microbial succession to lignocellulose transformation.

#### 3.6.1. Dynamic Abundance Profiles of CAZy Families

CAZyme-encoding gene abundance generally increased with compost maturation, with pronounced enrichment at the thermophilic (day 15) and maturation (day 30) stages (Figure 6B). In MP, Glycoside Hydrolase (GH), Auxiliary Activity (AA), and Carbohydrate Esterase (CE) families associated with lignocellulose degradation remained relatively abundant. GH families, primarily involved in cellulose and hemicellulose hydrolysis, were more abundant in MP, indicating enhanced genetic potential for polysaccharide depolymerization [40,41]. IB showed greater fluctuations, with cellulolytic families such as GH29 and GH92 increasing transiently before declining. RI showed slightly weaker lignocellulose-related CAZy enrichment than MP, whereas CK had lower overall CAZy-annotated gene abundance and weaker coordination among carbohydrate-degradation functions.

#### 3.6.2. Contribution of Functional Microorganisms to Carbohydrate Degradation

Functional contributions of major lignin-, cellulose-, hemicellulose-, and chitin-degrading genera showed clear temporal succession. On day 0, *Thermobifida*, *Thermopolyspora*, and *Cellvibrio* were rare (<0.001), whereas *Corynebacterium*, *Pseudomonas*, *Jeotgalibaca*, and *Fundicoccus* dominated, indicating limited initial lignocellulose-degradation potential. At the thermophilic stage (day 15), lignocellulose-related genera showed increased contributions. *Thermopolyspora* contributed to lignin/cellulose (0.086) and hemicellulose (0.088) degradation in IB and chitin degradation in MP (0.073). *Thermobifida* contributed to lignin degradation in IB (0.075), cellulose and hemicellulose degradation in RI (0.078), and chitin degradation in CK (0.113), while *Cellvibrio* contributed mainly in CK (0.006–0.010). On day 30, contributions remained treatment-dependent. *Thermobifida* (0.018–0.061), *Thermopolyspora* (0.013–0.043), and *Cellvibrio* (0.031–0.040) remained important in RI, whereas *Cellvibrio* contributed strongly in IB (0.060–0.074). These patterns demonstrated treatment-specific microbial contributions to continuous carbohydrate degradation.

On day 30, *Xiashengella* showed high abundance and functional contribution across treatments. Unlike *Thermobifida*, *Thermopolyspora*, and *Cellvibrio*, it was associated with the utilization of residual oligosaccharides, monosaccharides, and partially degraded polysaccharides, suggesting a role in late-stage carbohydrate turnover after macromolecule breakdown [42]. This pattern indicated complementary functions between primary degraders and late-stage carbohydrate-utilizing taxa during compost maturation (Figure 6C).

Figure 6D showed relatively low overall functional abundance on day 0, indicating limited initial lignocellulose-degradation potential. CAZy-annotated functional abundance increased markedly by day 15; cellulase reached 4273.99 in MP, and hemicellulase reached 8938.78 in IB. On day 30, hemicellulase and cellulase remained abundant in MP at 8899.79 and 4897.36, respectively, while cellulase reached 5262.47 in CK. Although CK showed high cellulase abundance, MP maintained more balanced enrichment of CAZy-annotated genes associated with cellulase and hemicellulase functions during maturation [43].

An integrated Sankey diagram summarized normalized linkages among physicochemical factors, microbial communities, and carbon-transformation indicators (Figure S3), with flow width representing the relative weight at each level. Compared to CK, MP had a higher weight for the physicochemical environment (11.07% vs. 9.15%), together with higher weights for bacterial and fungal communities and key genera such as *Pseudoxanthomonas* and *Aspergillus*. Weights associated with organic carbon substrates and humic carbon fractions increased from 11.59% to 14.02% and from 4.43% to 5.36%, respectively. These patterns associated the Fe_3_O_4_ treatment with coordinated changes in the composting environment, microbial succession, organic matter transformation, and humification.

## 4. Discussion

Distinct iron-based amendments regulated the aerobic co-composting of *P. citrinopileatus* SMS and chicken manure through different physicochemical and biological pathways. MP provided the most balanced performance in lignocellulose conversion, organic matter transformation, humification, and compost maturity. RI favored hemicellulose degradation and humic substance accumulation, whereas IB accelerated early heating and showed relatively high cellulose degradation but greater physicochemical fluctuations. These responses likely reflected differences in composition, solubility, surface properties, and redox reactivity.

### 4.1. Effects of Iron-Based Amendments on Compost Physicochemical Properties and Humification

The three iron-based amendments differentially affected compost conditions, organic matter transformation, and humification. MP showed the most balanced performance, sustaining thermophilic conditions while achieving a relatively low EC, the highest final GI, the lowest final OM content, and a low final C/N ratio. These characteristics indicated advanced compost maturity and extensive organic matter transformation. Similar enhancements in lignocellulose degradation and humification following magnetite addition have been reported during composting [44]. In contrast, IB produced the fastest initial heating but greater fluctuations in temperature, pH, EC, and nitrogen forms. Its low early-stage GI indicated transient phytotoxicity, while a secondary temperature increase during maturation suggested renewed decomposition. However, its relatively high final OM content indicated less extensive transformation than in MP and RI (Figure 1B, Figure 2A–C and Figure 3A). RI showed the highest hemicellulose degradation rate (Figure 3B and Table 2) and greater final accumulation of HA and total humic substances (Figure 3A). These responses indicated that Fe_2_O_3_-rich crude iron ore preferentially promoted hemicellulose transformation and humification, although its overall lignocellulose degradation was lower than that of MP. Overall, Fe^0^ beads accelerated early heating but reduced process stability, whereas Fe_2_O_3_-rich crude iron ore favored hemicellulose conversion and humification.

### 4.2. Linkages Between Lignocellulose Conversion and Microbial Functional Potential Under Iron-Based Amendments

MP achieved the highest lignin degradation rate among the treatments (Figure 3B and Table 2), possibly through coupled abiotic and microbial processes. The mixed-valence Fe^2+^/Fe^3+^ structure of Fe_3_O_4_ can facilitate electron transfer and, with microbially derived H_2_O_2_, generate Fenton-like reactive oxygen species that partially oxidize lignin and improve its accessibility to microbial enzymes [44,45]. Consistently, *Aspergillus* and *Penicillium* were enriched in MP during the thermophilic stage, while functional contribution analysis associated *Thermobifida*, *Thermopolyspora*, and *Cellvibrio* with annotated lignin-degradation functions (Figure 4B and Figure 6C). Relevant enzymes include fungal laccases, lignin peroxidases, and manganese peroxidases [45], as well as bacterial DyP-type peroxidases; a characterized enzyme from Thermobifida fusca can oxidize Kraft lignin and lignin-model compounds [46]. The relatively high abundance of AA-family genes in MP (Figure 6B) further supported oxidative lignin modification because the CAZy AA class includes ligninolytic redox enzymes and auxiliary oxidoreductases [47]. GH and CE families may subsequently hydrolyze cellulose and hemicellulose exposed following lignin disruption. Thus, Fe_3_O_4_ may promote lignin conversion by coupling iron-mediated oxidation with microbial enzymatic degradation, contributing to the higher total lignocellulose degradation in MP (Figure 3B and Table 2).

Treatment comparisons showed that high cellulose degradation did not necessarily indicate efficient deconstruction of the entire lignocellulosic matrix. In CK, readily accessible cellulose may have been preferentially utilized by a comparatively less rich but functionally active community. The presence of *Thermobifida* and *Cellvibrio* and the persistence of cellulase-related genes supported cellulose hydrolysis without iron addition (Figure 4B and Figure 6B,C). However, limited lignin removal may have restricted access to cellulose embedded in the lignin–carbohydrate matrix, constraining overall conversion relative to MP (Figure 3B and Table 2). IB also promoted early heating and cellulose utilization but showed greater physicochemical and microbial fluctuations (Figure 1B, Figure 2A and Figure 4A,B). In contrast, RI was associated more closely with hemicellulose conversion and humic substance accumulation than with extensive lignin removal (Figure 3A,B; Table 2). Thus, different iron forms regulated substrate conversion through amendment-specific physicochemical and microbial pathways.

MetaCyc analysis provided complementary evidence of amendment-specific downstream carbon metabolism. MP showed greater enrichment of fermentation-related genes, IB showed greater enrichment of genes related to hydrogen production and methanogenesis, and RI showed greater enrichment of genes associated with organic carbon and acetate oxidation (Figure 6A). Although these pathways do not directly represent ligninolytic activity, they reflect different routes for processing soluble and partially degraded carbon released during lignocellulose conversion. Together, lignin loss, microbial succession, CAZy profiles, and carbon-metabolism pathways support the hypothesis that Fe_3_O_4_ promotes lignin conversion by coupling iron-mediated oxidation with microbial enzymatic degradation. Future studies integrating reactive oxygen species quantification, iron-speciation analysis, and ligninolytic enzyme activity assays should test and refine this proposed mechanism.

### 4.3. Microbial Succession in Relation to Environmental Factors

Microbial succession closely tracked changes in compost physicochemical conditions and organic matter transformation. Spearman correlation, CCA, Mantel tests, and VPA identified the C/N ratio, nitrogen forms, OM, and humic fractions as major covariates of bacterial and fungal community structure (Figure 5A–C and Figure S2). Thermophilic and lignocellulose-associated taxa were associated with increasing TN, NO_3_^−^-N, and HA and decreasing OM and C/N ratios, consistent with roles in nitrogen transformation, organic matter decomposition, and humification. Conversely, early-stage taxa such as *Corynebacterium* and *Blastosporella* were associated with higher NH_4_^+^-N and C/N ratios and declined during maturation. These coordinated trends indicated a transition from communities utilizing readily available substrates to thermophilic and maturation-stage decomposers associated with lignocellulose transformation and humic substance formation [48,49].

The divergent overall Chao1 and Shannon patterns in MP reflected different dimensions of microbial α-diversity. Chao1 estimates taxon richness and is sensitive to rare taxa, whereas the Shannon index incorporates both richness and evenness. In MP, overall richness increased throughout composting, while the Shannon index increased during the early and thermophilic stages and then remained nearly stable, ending below CK and RI but above IB. Thus, MP supported a broad taxon pool with less even relative abundances. Sustained thermophilic conditions, relatively low EC, and the Fe^2+^/Fe^3+^-associated redox environment may have maintained rare taxa while favoring *Thermopolyspora*, *Luteimonas*, *Pseudoxanthomonas*, *Aspergillus*, and *Penicillium*. This combination of high richness and functional dominance explains the highest final Chao1 richness but intermediate Shannon index in MP. Together with the associations between lignocellulose-related taxa and TN, NO_3_^−^-N, HA, OM, and the C/N ratio, these patterns suggest that physicochemical filtering and substrate transformation jointly shaped microbial richness and evenness (Table S1; Figure 4A and Figure 5A–C).

## 5. Conclusions

This study demonstrated that the three iron-based amendments exerted distinct effects on the aerobic co-composting of *P. citrinopileatus* SMS and chicken manure. Fe_3_O_4_-based magnetic powder provided the most balanced overall performance by promoting lignocellulose conversion, organic matter transformation, humification, process stability, and compost maturity. Fe_2_O_3_-rich crude iron ore preferentially enhanced hemicellulose conversion and humic substance accumulation, whereas Fe^0^ beads accelerated early heating and cellulose degradation but caused greater process fluctuations. Metagenomic and environmental association analyses indicated that these treatment-dependent outcomes were accompanied by distinct microbial succession, functional potential, and carbon-metabolism profiles, supporting an integrated relationship among iron form, compost conditions, microbial functions, and substrate transformation. From an application perspective, Fe_3_O_4_-based magnetic powder should be prioritized when balanced composting performance is required. Fe_2_O_3_-rich crude iron ore may be considered when hemicellulose conversion and humification are the primary objectives, whereas Fe^0^ beads may be less suitable as a first choice when process stability is prioritized. Further pilot-scale studies should evaluate amendment dosage, economic feasibility, environmental safety, and material recovery or reuse.

## Figures and Tables

**Table 1:** Treatment composition and basic characteristics of the iron-based amendments used in the composting experiment.

Treatment Group	Total Moisture Content	Dry Matter Content: 40%				Total Wet Weight (kg)
		*P. citrinopileatus* SMS	Chicken Manure	Iron-Based Amendment		
				Main Component	Particle Size	
CK	60%	30%	10%	—	—	50
RI		29.1%	9.7%	Fe_2_O_3_-rich crude iron ore	≤2 mm	
MP				Fe_3_O_4_-based magnetic powder		
IB				Fe^0^ beads		

Note: In the amended treatments, the corresponding iron-based amendment accounted for 3% of the total dry matter, equivalent to 1.2% of the total wet mass. The remaining dry matter consisted of *P. citrinopileatus* SMS and chicken manure at a dry-weight ratio of 3:1. Before use, all three iron-based amendments were separately ground and passed through a 2 mm sieve to obtain particles ≤2 mm.

**Table 2:** Degradation rates (%) of lignocellulosic components after 30 days of composting under different treatments.

Lignocellulosic Component	CK	RI	MP	IB
Lignin	38.01%	42.98%	52.69%	40.52%
Cellulose	85.55%	51.83%	74.42%	84.11%
Hemicellulose	66.20%	78.96%	77.88%	60.05%
Total lignocellulose	49.18%	51.45%	59.48%	48.82%

Note: Degradation rate (%) was calculated as [(C_0_ − C_30_)/C_0_] × 100, where C_0_ and C_30_ represent the treatment mean contents of each lignocellulosic component on days 0 and 30, respectively.

## Data Availability

The original contributions presented in this study are included in the article and Supplementary Materials. Further inquiries can be directed to the corresponding author.

## Supplementary Materials

The following supporting information can be downloaded at https://www.mdpi.com/article/10.3390/foods15173158/s1, Table S1: Overall α-diversity indices calculated from the combined bacterial and fungal taxonomic profiles of compost samples; Figure S1. Phylum-level succession of fungal and bacterial communities during 30-day composting under different treatments; Figure S2. Genus-level variation partitioning analysis (VPA) of three environmental factor groups explaining bacterial (left) and fungal (right) community variation; Figure S3. Sankey diagram of carbon metabolism contribution pathways under different iron-based material treatments (CK, RI, MP, IB).
