# Supplementary material for: Iron-Amended Pleurotus citrinopileatus Residue Composting: Lignocellulose Degradation and Microbial Functional Potential
Source: Foods. 2026 Sep 6;15(17):3158. doi: 10.3390/foods15173158 (PMC13564553; doi:10.3390/foods15173158)
Supplement: Supplementary file 1 [file foods-15-03158-s001.zip › foods-4500511-supplementary.pdf]

Supplementary Materials

Table S1. Overall α-diversity indices calculated from the combined bacterial and fungal taxonomic profiles of compost samples.

Sample	Chao 1	Shannon	Simpson
CK_0	18472	5.48	0.01
CK_15	31306	6.69	0.01
CK_30	31523	6.95	0.01
RI_0	17229	5.32	0.02
RI_15	32101	6.97	0.01
RI_30	32185	6.89	0.01
MP_0	20085	5.30	0.02
MP_15	30954	6.78	0.01
MP_30	32659	6.79	0.01
IB_0	18294	5.31	0.02
IB_15	29878	6.31	0.01
IB_30	31096	6.70	0.01

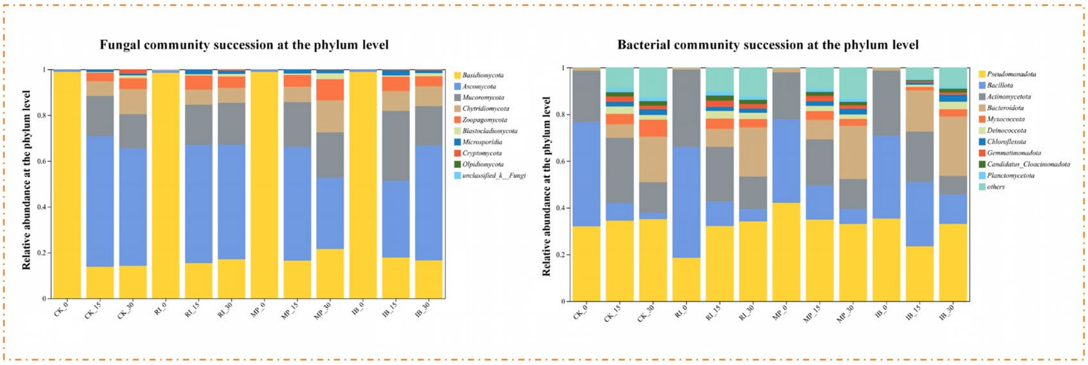

Figure S1. Phylum-level succession of fungal and bacterial communities during 30-day composting under different treatments. Stacked bar charts show the relative abundances of dominant fungal phyla (left) and bacterial phyla (right) in samples collected on days 0, 15, and 30. Low-abundance bacterial taxa are grouped as “others”.

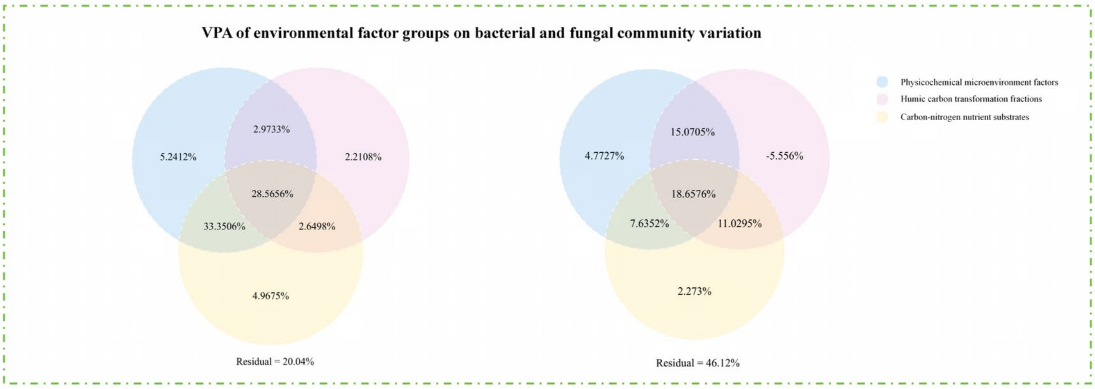

Figure S2. Genus-level variation partitioning analysis (VPA) of three environmental factor groups explaining bacterial (left) and fungal (right) community variation. Non-overlapping and overlapping areas represent the unique and shared fractions of explained variation, respectively, whereas the residual represents the unexplained variation. Negative fractions may occur when adjusted (R^2) values are used and were retained without truncation. The three environmental factor groups comprised physicochemical microenvironment factors (pH, EC, temperature, GI, AP, and AK), humic carbon transformation fractions (HS, HA, FA, and HM), and carbon–nitrogen nutrient substrates (NH_4^+-N, NO_3^--N, TN, TC, C/N ratio, and OM).

Sankey diagram of carbon metabolism contribution pathways under different iron-based material treatments

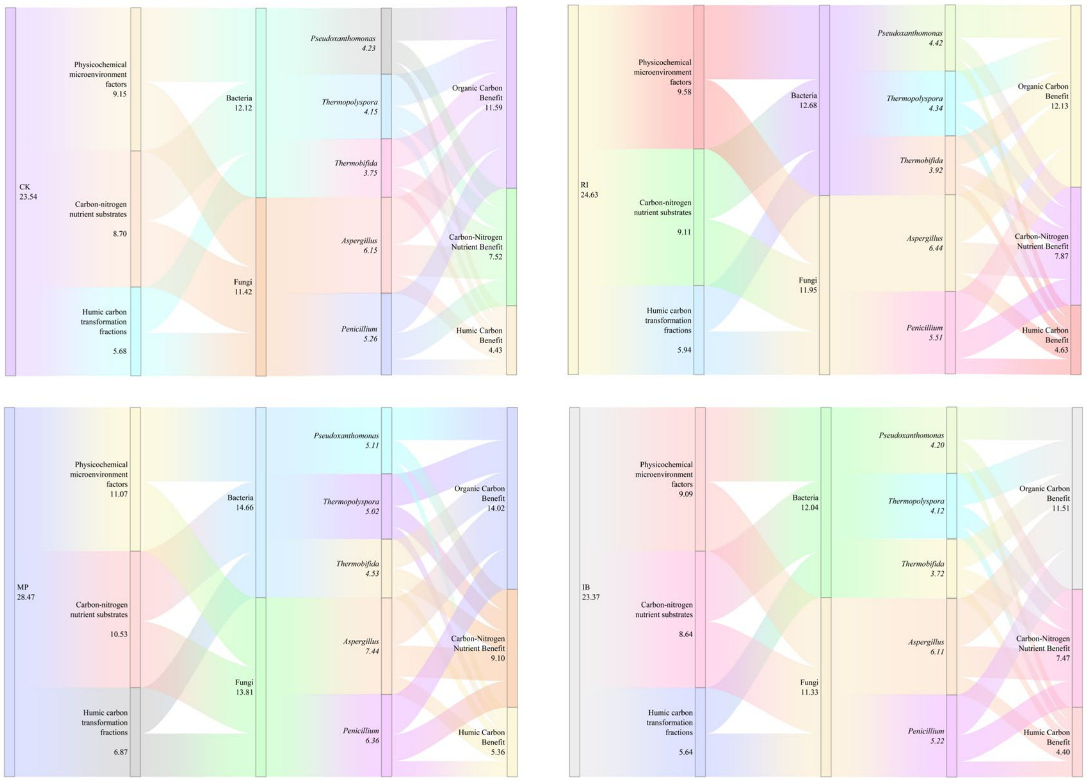

Figure S3. Sankey diagram of carbon metabolism contribution pathways under different iron-based material treatments (CK, RI, MP, IB). This diagram illustrates the contribution flow of environmental factors-microbial communities-carbon metabolism benefits. The flow width is proportional to the normalized contribution value of the corresponding level. Node values are the relative contribution ratios normalized based on the total amount of each level, rounded to two decimal places. Due to software display precision limitations, the sum of data at each level may have a minor deviation of ±0.02%, while the actual data are fully conserved. Level 1: Total carbon metabolism contribution of different treatments, normalized by total lignocellulose degradation rate. Level 2: Three driving factors (defined in Figure S2), normalized by total VPA explanation of bacteria and fungi. Level 3: Bacteria and fungi, normalized by CCA explanation of bacteria, fungi and environmental factors. Level 4: Dominant bacterial and fungal genera, normalized by R-value of dominant microbial abundance. Level 5: Three functional benefits, normalized by R-value of dominant microbial abundance. Carbon-nitrogen nutrition benefits include TN, NH_4^+-N, NO_3^--N, C/N; organic carbon substrate benefits include Temperature, pH, EC, AK, AP, GI, TC, OM; humus carbon benefits include HS, HA, FA, HM.
